# Pahon Cave, Gabon: New insights into the Later Stone Age in the African rainforest

**DOI:** 10.1371/journal.pone.0336405

**Published:** 2025-12-10

**Authors:** Marie-Josée Angue Zogo, Isis Mesfin, Geoffroy de Saulieu, Wim Van Neer, Els Cornelissen, David Pleurdeau, Richard Oslisly

**Affiliations:** 1 UMR 208 PALOC-IRD-MNHN-CNRS, Paris, France; 2 UMR 7194 HNHP/MNHN/UPVD- Institut de Paléontologie Humaine, Paris, France; 3 Cellule scientifique de l’Agence Nationale des Parcs Nationaux du Gabon (ANPN), Libreville, Gabon; 4 Royal Belgian Institute of Natural Sciences, Brussels, Belgium; 5 Laboratory of Biodiversity and Evolutionary Genomics, University of Leuven, Leuven, Belgium; 6 AfricaMuseum, Tervuren, Belgium; Sapienza University of Rome: Universita degli Studi di Roma La Sapienza, ITALY

## Abstract

Although the Later Stone Age as a distinctive techno-cultural phase has disappeared, forager groups in the African rainforest persist today. However, their origins remain poorly understood. The absence of stone tool production raises questions about the pace and processes of its decline and its relationship to the emergence or adoption of metallic tools. Archaeological sequences from the Middle and Late Holocene are particularly valuable for documenting the coexistence of diverse subsistence strategies and technologies within the Central African rainforest. In this context, the Pahon Cave sequence, in Gabon, spanning a period from 7,571 cal. BP to 2,523 cal. BP, provides an opportunity to study the evolution of stone tool production in the rainforest of the Ogooué Basin. This chronological range coincides with significant broader techno-cultural and environmental changes in Central Africa. This article provides a detailed description of the lithic industry for each layer, along with the identification of faunal remains, giving insight into the exploitation of rainforest resources and hunting practices. At Pahon Cave, our findings suggest that stone tool technology remained stable over time, at least until around 2,523 cal. BP. Furthermore, the technological characteristics of the lithic industry indicate no clear cultural affiliations. These features contribute highlighting a techno-cultural diversity during the Middle and Late Holocene Later Stone Age in Atlantic Central Africa.

## 1. Introduction

### 1.1 Middle to Late Holocene in Central Africa: A diversity of lifestyles?

In Central Africa, the Late Holocene (from 4,250 BP) is marked by significant cultural changes. Based on the different terminologies used by researchers, these include the Later Stone Age (LSA), a ‘Neolithic phase’ with polished stone tools, a lithic industry based on flake production, and ceramics, followed by the Iron Age, which emerged between at minimum 2,800 and 2,600 BP in Central Africa [1,2].

The LSA may have developed as early as 40,000 BP in Atlantic Central Africa [3], but its end remains uncertain in Central Africa. Indeed, forager groups in Central Africa did not disappear but eventually abandoned stone tool technology [4], we don’t know when and how.

In addition, it seems that during the Late Holocene LSA various subsistence strategies coexisted, reflecting diverse traditions, particularly marked by pottery use and, in some cases, metalworking. In this regional archaeological context, archaeological sites with lithics, no metal, and no pottery are usually considered as LSA. While the precise timing and origins of agriculture in Central Africa remain unclear, it has been suggested that this practice may have appeared as early as the Middle Holocene (8,236 à 4,250 years BP) at Shum Laka rock shelter, Cameroon [5]. During this period, basalt macrolithic tools are found associated with pottery and botanical remains (e.g., *Canarium schweinfurthii*); suggesting increasingly specialized gathering practices.

The Late Holocene (from 4,250 BP) is thus a crucial and complex period in the history of populations in Atlantic Central Africa, both culturally and biologically [5–7]. At this time, the Bantu expansion is already being discussed. The Bantu expansion is a hypothesis that initially emerged from studies in historical linguistics. The vast majority of languages spoken from the Cameroon/Nigeria border to the Indian Ocean and the Cape of Good Hope are related and are thought to derive from a Proto-Bantu language (see summary in Bostoen 2015 [8]. With the rise of DNA studies, it has become clear that this linguistic expansion was accompanied by a demographic expansion [9–11]. Archaeological data [12–14] reveal a major shift in population dynamics, with the establishment of sedentary farming villages with pottery, visible around 2800 BP. Thus, it is generally assumed that the Bantu expansion may have begun as early as 3000 BP.

However, the coexistence of different subsistence strategies in Central Africa during this time has been questioned by archaeologists, whereas it challenges linear models of cultural evolution. In addition, the development of DNA studies highlighting complex scenario among past hunter-gatherers groups [15] can still barely rely on archaeological data, making any archaeological sequence documenting this time period particularly important.

In this context, the Late Holocene in Central Africa presents itself as a cultural diversity in which the role and status of the last LSA groups remain poorly understood: how were they impacted by these transformations? In what cultural and ecological context did the Bantu expansion take place? To what extent this context is auspicious to these cultural changes during Late Holocene?

### 1.2 Pahon Cave site setting

At Pahon Cave (12°45’15“E/0°48’42”S) in Gabon, the archaeological sequence provides an opportunity to shed light on LSA groups during the critical period spanning the Middle and Late Holocene. This well-preserved, finely stratified sequence contains both lithic and faunal remains in a guano-based sediment [16]. The presence of finely bedded, horizontally continuous guano layers—clearly visible in the stratigraphic illustration and in both test pits—suggests primary deposition in a stable environment [17].

**Fig 1:**
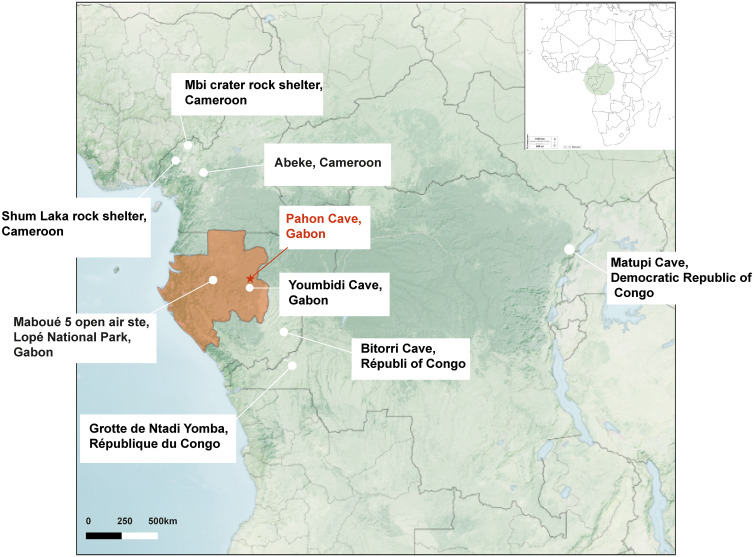
Map of Central Africa and the sites mentioned in the text. This Map shows the distribution of sites contemporany to Pahon sites. Map modified from https://earthobservatory.nasa.gov/map#5/-0.644/25.567.

Pahon Cave is located in the Ogooué-Lolo Province, within the municipality of Lastoursville Fig 1. The region is karstic, characterized by massive dolomitic rock formations dating to the Lower Proterozoic (- 2000–1900 million years) [18]. It lies in the central-eastern part of Gabon’s dense, evergreen rainforest, dominated by persistent-leaf tree species typical of regions with high and consistent rainfall throughout the year [19]. The cave is adjacent to Youmbidi Cave, which is currently being excavated and contains a contemporary archaeological layer [20].

**Fig 2:**
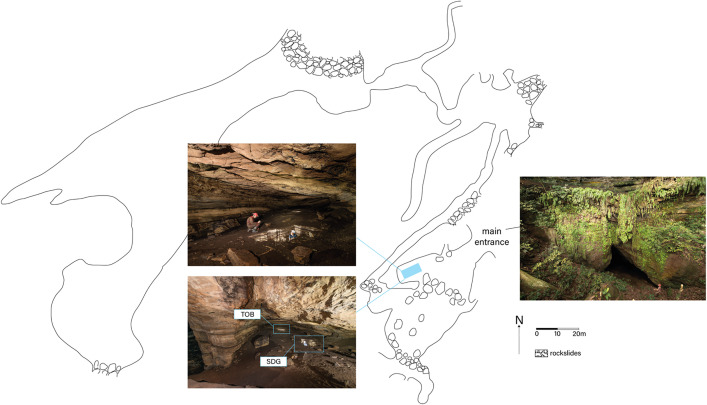
Pahon Cave. View of Pahon Cave entrance where has been excavated the two trenches – © Photos from Serge Caillault.

Gabon is largely covered by tropical rainforests, which account for approximately 85% of its territory [21]. Around Lastoursville, nearly 50 caves were located by teams from the Ogooué Mining Company (Comilog) between 1976 and 1978 [22] and by the National Parks Agency (ANPN) between 2013 and 2015 [23]. These caves have been included on UNESCO’s Tentative World Heritage List since 2005. The Pahon Cave Fig 2. which extends for over 1,100 meters, opens at the base of a cliff through an impressive main entrance. It continues along a steep scree slope into a vast chamber with ceiling heights exceeding 20 meters, connected by a low gallery to other, more distant and even larger chambers. The excavation area is located in the penumbra, 50 meters from the entrance, on a small platform overlooking a stream.

**Fig 3:**
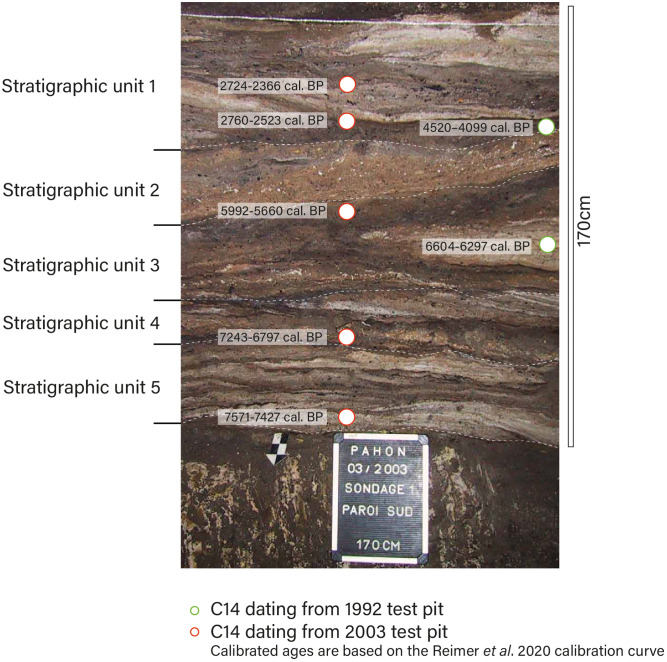
Statigraphic profile of the Pahon Cave. Stratigraphy and chronology of the profile of SDG test-pit of Pahon Cave @ R. Oslisly.

In 1992, a test excavation was conducted [16], and in 2003, two additional test excavations were carried out in Pahon Cave: TOB test-pit was 1x1m and SDG test-pit was 1x3m Fig 3.

**Fig 4:**
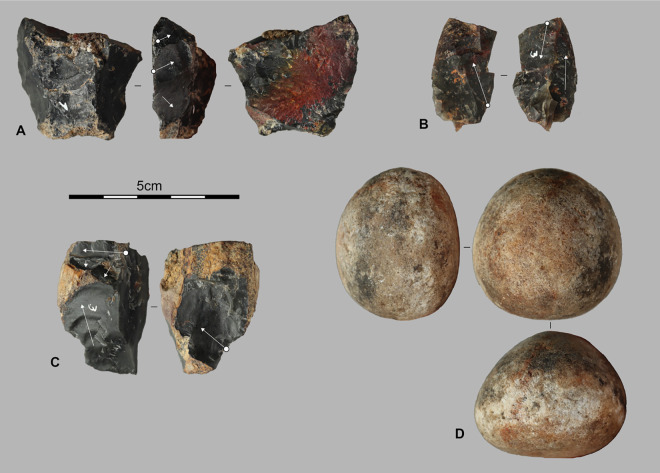
Pottery shards. Pottery sherds found on the surface of the Pahon Cave.

A total volume of 4.82 m³ was excavated. Excavation was carried out following 10 cm spits, and all material was dry sieved on a 4 mm mesh. The two test excavations at Pahon Cave produced a significant quantity of lithic material, along with archeozoological and archaeobotanical remains. Pottery sherds were recovered only on the surface Fig 4.

Despite the small size of the excavated areas, a few refitting units could be identified (E.C.). Three simple refits involve only 7 chert artifacts from the SDG trench and 2 siliceous rock artifacts from the TOB test pit. The refits remain within a single level (−70–80 cm and −80–90 cm for SDG, below the concentration, and 80–100 cm for TOB, within the concentration). In SDG, this consists of a group of three fragments from a core displaying centripetal exploitation on the upper face and a lower cortical. A flake on a nucleus fragment and two flakes are associated with the initial decortication phase of a material that exhibits multiple small and large cleavage planes as well as cortical surfaces. In TOB, one *débris* and a core fragment place this refit within the preparing phase of a core.

This cave contains a well-preserved stratigraphic sequence, yielding dates from the Middle to Late Holocene, ranging between 2,724–2,366 cal BP and 7,571–7,427 cal BP. These dates first appeared in the article by Oslisly et al in 1994 [16]. All C14 dates have been made on charcoals at the dating laboratory in Gif-sur-Yvette, France. Charcoal remains were collected in situ in the profile section. We calibrated the dates using the Calib software [24]. This chronology implies that human populations inhabited this cave during and after the “African Humid Period,” which occurred between 14,800 and 5,500 BP [25,26] and was characterized by more abundant rainfall and the expansion of tropical forests; but also during significant regional changes including ceramic traditions emergences and change in foraging strategies and finally, the Bantu Expansion.

### 1.3 Pahon Cave’s contribution to stone age studies and regional LSA sites

The significance of Pahon Cave lies in its finely preserved stratigraphy and archaeozoological remains, which provide valuable insights into the LSA of the equatorial forests of Gabon. Archaeobotanical analyses from the site revealed pollen from several species, the most frequent being *Ceiba pentandra*, commonly known as the *kapok* tree. This species, often found in peri-forest savannas, is an indicator of semi-deciduous forest environments [16].

The number of archaeological sites in Central Africa with faunal preservation is extremely limited due to the region’s acidic soils [27]. Typically, animal remains are found in caves or rock shelters, whereas open-air sites only preserve faunal remains under specific conditions and are generally more recent. Preservation is often facilitated by rapid and deep burial, such as in riverine sites of the Inner Congo Basin [28]. On coastal sites, bones can better withstand acidic conditions when sediment is tempered by the presence of sea shells [29]. In Gabon, faunal remains have so far been reported from Iron Age sites like Toubé-1 and Okanda-5 [30], the Neolithic site of Otoumbi-13 [31], and Pahon Cave. The mid-Holocene faunal assemblage from Pahon Cave has significant potential to enhance our understanding of subsistence strategies during a poorly documented period and of the ecological history of the rainforest in Africa.

Central Africa has very few archaeological sites with sequences capable of documenting the transitions that occurred during the Middle Holocene. Of these, only a handful have published faunal remains, such as Ntadi Yomba [32]. Consequently, Pahon Cave stands out as one of the rare sites in Central Africa with a well-preserved sequence that includes faunal, lithic, and archaeobotanical remains. The chrono-cultural context of Pahon makes it comparable with the following regional sites:

- **Shum Laka rock shelter, Cameroon** Fig 1 [33–35]:

Shum Laka is one of Central Africa’s most significant archaeological sites, located at the base of a cliff in a caldera at 1,650 m asl, behind a waterfall. Covering a total area of about 1,200 m², the site’s sequence spans from the late Pleistocene to the modern era, with the oldest dates at 38,406–34,591 cal BP. The site recorded an important part of the technocultural evolution, including lithic remains (mainly quartz and later basalt) attributed to the LSA, ceramics, faunal (including several primate taxa) and archaeobotanical remains, and iron objects in the more recent layers. A total of 18 human skeletons have been discovered at Shum Laka, comprising 2 distinct burial phases [15,33,36,37].

- **Bitorri Cave, Republic of Congo** Fig 1 [38]:

This site is located within a schist-limestone formation and comprises a cave approximately 100 m deep, with an entrance 10 m wide and 2–3 m high. A horizontal excavation revealed 20 archaeological levels with lithic tools. The uppermost layer contains pottery and iron objects, with dates ranging from 3,959 to 5,049 cal BP.

- **Ntadi Yomba Cave, Republic of Congo** Fig 1 [39,40]:

Situated on a schist-limestone hill, this rock shelter is 10 m long and 4 m deep. It yielded lithic materials attributed to the Tshitolian [31,32], ceramics, and faunal remains, including black rhinoceros. The faunal remains fall into two categories: animals that died naturally or were brought by other predators, and kitchen waste from prehistoric humans. The site has levels dated between 1703–2132 cal BP and 7748–8015 cal BP.

- **Youmbidi Cave, Gabon** Fig 1 [20]:

This rock shelter near Pahon in Lastoursville is currently under excavation, with several dates ranging from 958–1060 cal BP to 28,874–29,263 cal BP (from a 2019 excavation). The lithic materials are associated with archaeobotanical and archaeozoological remains and minimal ceramics in the uppermost layer.

- **Mbi Crater rock shelter, Cameroon** Fig 1 [41]:

Located at 2,500 m asl, this volcanic and marshy crater includes a large rock shelter. Lithic materials attributed to the LSA, numerous faunal and archaeobotanical remains, and human remains dated to 8,386–8,777 cal BP have been discovered. The sequence also includes dates ranging from 2,745–3003 cal BP to 2,484–2,929 cal BP.

- **Abeke, Cameroon** Fig 1 [42]:

This rock shelter spans a total area of 3,265 m², with three stratigraphic layers containing lithic materials and some faunal remains. The site has been dated to 6,169–6,629 cal BP and shows a gradual transition from LSA lithic industries to larger, polished tools.

- **Matupi Cave, Democratic Republic of Congo** Fig 1 [43–46]:

A dolomite cave in the Mont Hoyo massif, Matupi measures 7 m in height, 5 m in width, and 8 m in depth. Excavated remains include lithic materials (mainly microlithic technology on quartz), faunal and archaeobotanical remains, and iron artifacts. The site’s dates range from 42,179–34,475 cal BP over a depth of 195 cm.

- **Maboué 5 open air site, Lopé National Park, Gabon** Fig 1 [47]:

The most artifact-rich level, dated between 44,600–42,431 cal BP, features industries resembling both MSA and LSA, including quartz flakes, scrapers, burins, drills, bifacial fragments, and leaf-shaped points. The level dated to 5,280–4,860 cal BP is contemporaneous with Pahon’s sequence but yielded fewer materials [3,48].

## 2. Materials and methods

### 2.1 Lithic assemblage

The lithic material originates from the TOB and SDG excavations conducted in 2003, totaling 1,113 artifacts. The assemblage exhibits a high rate of fractures and knapping accidents (Siret fractures and transverse fractures), primarily linked to the fissure planes of chert blocks—a rock naturally found in the caves of Lastoursville and its surroundings. Four main types of raw materials were identified: quartz and dolomit in small quantities (n = 298) and (n = 106), a few rare flakes of quartz crystal (n = 24), and, most abundantly, chert (n = 733), which appears in black or gray with varying levels of transparency. This chert, despite natural fissures, is conducive to knapping, as the conchoidal fracture marks are easily discernible.

For the TOB excavation, within the first meter of sediment, the artifacts were recorded in layers of 10 cm except for the last spit that is 20 cm thick due to lack of time during fieldwork: 30–40 cm; 50–60 cm; 60–70 cm and 80–100 cm.

Several sedimentary layers did not deliver artefact (40–50 cm and 70–80 cm), suggesting distinct phases of occupation within the cave. A total of 338 artifacts, primarily from the lower layers (below 80 cm), make up the corpus of the TOB excavation Table 1.

**Table 1:** Techno-typological details of TOB Trench from Pahon Cave.

Lithic types	10-20 cm	30-40 cm	50-60 cm	60-70 cm	80-100 cm	Total (n=)	Total (%)
**Simple flakes**	2	33	30	14	44	123	31.7%
**Elongated flakes and blades**	0	10	11	0	38	59	15.2%
**cortical and semi-cortical flakes**	3	14	7	14	30	68	17.5%
***Débris***	0	1	1	0	20	22	5.7%
**Fragments**	0	4	3	6	15	28	7.2%
**Retouched pieces**	0	1	2	1	6	10	2.6%
**Cores**	0	0	3	5	15	23	5.9%
**Core-edge/ *Débordant* flakes**	1	4	8	9	18	40	10.3%
**Naturals**	0	4	0	9	1	14	3.6%
**Hammerstones**	0	0	0	0	1	1	0.3%
**Total**	**6**	**71**	**65**	**58**	**188**	**388**	100%

The SDG excavation, located 5 meters from TOB, yielded a total of 773 artifacts Table 2. Radiocarbon dating is only available for the SDG excavation Fig 3, and their stability confirms the site’s stratigraphic preservation. The artifacts are in the same state of preservation as those from TOB. The material is divided into chrono-stratigraphic unit (SU) 1–5, which consist of several sediment layers. This classification is based on both the natural sedimentary layers and the results of the radiocarbon dating. We have the following chrono-stratigraphic unit:

**Table 2:** Techno-typological details of SDG Trench from Pahon Cave per chronological Stratigraphic Unit (SU).

Lithic types	SU2	SU3	SU4	SU5	Total	Percent
**Simple flakes**	7	125	10	10	152	20%
**Elongated flakes/blades**	6	97	6	26	135	17%
**Cortical and semi-cortical flakes**	9	159	22	23	213	28%
***Débris***	1	34	4	0	39	5%
**Fragments**	7	41	14	6	68	9%
**Retouched pieces**	0	10	3	1	14	2%
**Cores**	0	21	1	2	24	3%
**Core-edge/ *Débordant* flakes**	1	66	1	12	80	10%
**Natural**	2	39	1	6	48	6%
**Total**	**33**	**592**	**62**	**86**	**773**	**100%**

- Stratigraphic Unit 1 (0–17 cm) (absence of lithic material) dated to 2,724–2,366 cal BP/ 2,760–2,523 cal BP/ 4,520–4,099 cal BP
- Stratigraphic Unit 2 (17–30 cm)
- Stratigraphic Unit 3 (30–80 cm) dated between 6,604–6,297 cal BP and 5,992–5,660 cal BP
- Stratigraphic Unit 4 (80–96 cm) dated to 7,243–6,797 cal BP
- Stratigraphic Unit 5 (96–120 cm) dated to 7,571–7,427 cal BP

### 2.2 Lithic analysis method

The material was sorted by excavation layer, and within each layer, we further classified the artifacts by type of lithic products Tables 1 and 2 in order to examine the techno-morphological characteristics of the lithic remains from the Pahon sequence. This involved determining the operational schemes, knapping methods, and performing tool types classification. We mainly followed the nomenclature of Inizan and colleagues [49,50]. We measured the length, width, and thickness according to the flaking axis. Within the flakes, we categorized the pieces as follows:

- Primary flakes or those with dorsal cortical surfaces, because these products provide information on the potential preparation of the cores and inform on the type of material (e.g., pebbles, blocks, slabs) and the early stages of knapping sequences.
- ‘Core-edge flakes’ also called ‘*débordant*’ flakes because they provide information on the surfaces adjacent to the flaking surface: preparation type, previous flaking surfaces (e.g., orthogonal cores). These can be intentionally produced to create a lateral surface on the flake or to rejuvenate a knapping surface.
- Elongated flakes were distinguished when the length was approximately twice the width and when the lateral edges were parallel to sub-parallel. Elongated flakes generally indicate specific production or targeted flakes type when they appear in large numbers in assemblages. Moreover, elongated flakes or ‘blades’ are well-documented in lithic technology worldwide, making it easier to characterize this type of production in an assemblage. However, following lithic technology concepts, the absence of clear evidence for laminar knapping prevents us from classifying these elongated products as ‘blades.

In contrast, *débris* are defined here as small knapping products that cannot be linked to any specific stage of the *chaîne opératoire* [49]. Natural fragments are characterized by the absence of removal negatives, the lack of sharp edges, and, conversely, multiple fracture planes. Finally, fragments are considered lithic pieces that resulted from intentional knapping but whose fracture has removed most of the techno-typological information [3,51,52].

Core classification relies on the description, relationship, and function of the surfaces related to the core’s volume management, as proposed by several authors whom we consulted for the method [49,53–55]. In our case, we identified the types of blank (e.g., flake, block, nodule etc.) and knapping methods applied at Pahon Cave to determine the objectives in terms of flake types (size, morphology) and their consistency with the collected assemblage of flakes.

Finally, the retouch was localized and interpreted in order to determine the types of tools that these knappers sought to produce based on both techno-functional approach and technological approach [49,56–58]. We distinguished the macroscopic use-wears through observation of every edges relying on [59,60]. No detailed description of the macro use-wears were conducted but artefacts displaying damaged edges were sorted for further microscopic analysis.

## 3. Lithic analysis of the TOB test-pit

Lithic analysis results are provided per test-pit and per layer. Following this division, the results are presented per type of product.

### 3.1 80–100 cm spit

#### 3.1.1 The cores.

**Fig 5:**
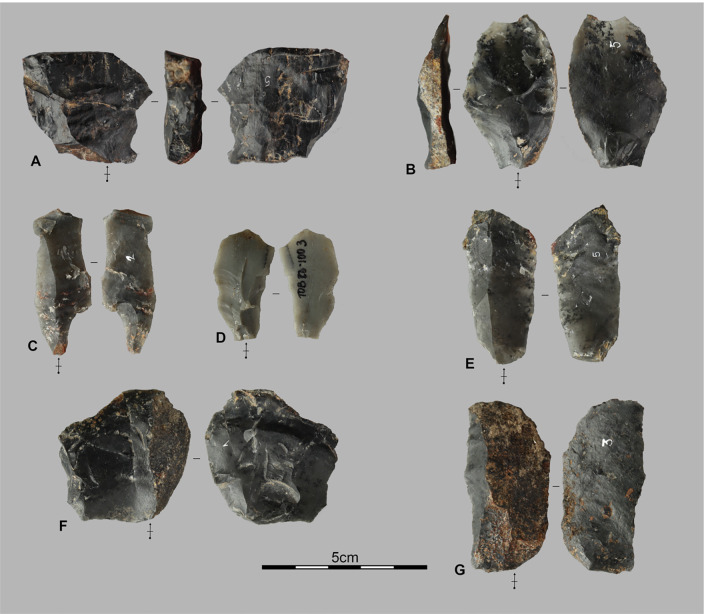
Lithic assemblages from the Pahon Cave. Artifacts from the 80–100 cm layer of the Pahon Cave; A and B are cores on flake, C is a core on a small block, and D is a hammerstone.

Among the 15 analyzed cores Fig 5, those on small blocks (n = 6) and pebbles (n = 2) are generally larger and heavier than those on flakes (n = 4). Block and pebble cores exhibit 4–6 multidirectional removals across 2–3 flaking surfaces, producing small flakes (<5 cm), predominantly short cortical or semi-cortical, without evidence of core preparation. These characteristics indicate brief, expedient reduction sequences.

In contrast, flake cores display 1–4 unidirectional to bidirectional removals. The resulting flakes are also small, morphologically varied, and lack cortex. Two flake cores bear bladelet negatives, and two present slightly prepared striking platforms. While all cores reflect direct freehand percussion, variations between the two groups likely relate to differences in blank type and intended flake production.

#### 3.1.2 Cortical and semi-cortical flakes.

**Fig 6:**
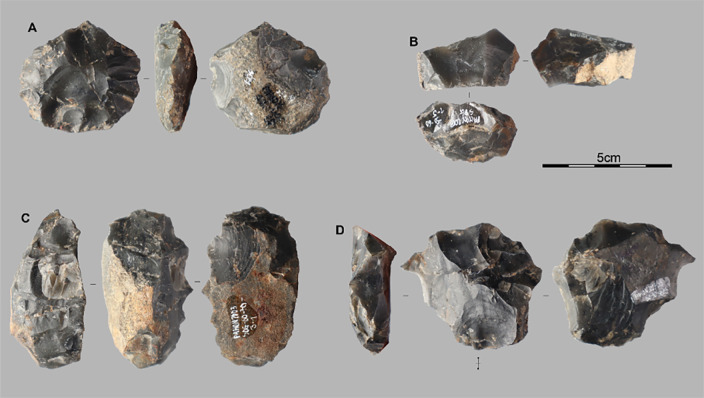
Lithic assemblages from the Pahon Cave. Artifacts from the 80–100 cm layer of the Pahon Cave; A and B are débordant flakes, C, D, E, and G are elongated flakes; F and G are semi-cortical flakes.

The primary flakes (n = 9) are mostly short and systematically smaller than 3 cm. They create very small openings on the initial blocks and appear consistently on the cores on blocks described above. In contrast, the semi-cortical flakes (n = 21) (examples F and G, Fig 6) exhibit more diverse sizes, ranging from small flakes (<3 cm) to medium ones up to 5 cm in length. Only two pieces show residual cortical surfaces on their distal part, while the others systematically display cortical surfaces on the lateral or latero-distal part. Platforms are mostly plain, suggesting an initial focus on opening striking platforms followed by a phase of lateral extensions of the flaking surfaces. This is also reflected in the predominantly unidirectional negatives of the semi-cortical flakes (including 4 semi-cortical flakes with bladelet negatives), which suggest unidirectional flaking phases.

#### 3.1.3 Débordant flakes.

*Débordant* flakes Fig 6 A and 6B represent 10.3% of the flake assemblage—a relatively high proportion. These small to medium-sized flakes remove a significant portion of the flaking surface, often spanning its full width. They may reflect surface reconfiguration or the intentional production of robust, functional flakes.

Most *débordant* flakes are cortical (n = 10), while others display plain dorsal surfaces or 2–3 predominantly unidirectional negatives. Dorsal surfaces lack cortex, with removals consistently unidirectional, suggesting standardized production. Five flakes exhibit macroscopic use-wear on sharp, fine cutting edges (cutting angles: 40°–60°, up to 75°), indicating potential functional use. Lateral surfaces, by contrast, may have facilitated gripping.

#### 3.1.4 Simple flakes.

Simple flakes are small to medium in size (≤5 cm long, ≤ 4 cm wide, ≤ 1.5 cm thick). Short flakes (length < width) are well represented (n = 7). Dorsal surfaces display 1–6 removal negatives, most often 2 (n = 13) or 3 (n = 8), with a dominant unidirectional orientation (n = 22), followed by bidirectional (n = 10) and multidirectional (n = 2). Despite this relative dorsal homogeneity, edge morphology remains highly variable. Striking platforms are typologically diverse, including cortical and semi-cortical (n = 10), dihedral (n = 8), plain (n = 9), and more rarely faceted (n = 2) or absent (n = 4), indicating variable preparation levels.

Macroscopic use-wear was observed on four flakes, characterized by unmodified lateral edges with cutting angles between 40° and 50°. These resemble the worn edges of *débordant* flakes but lack defined backs or gripping zones, suggesting opportunistic use.

#### 3.1.5 Elongated flakes.

Elongated flakes (Fig 6C–6E) are small to medium in size (3–6 cm long, ≤ 3 cm wide, > 1.5 cm thick), with average dimensions of 3.6 × 1.8 × 0.7 cm. Dorsal surfaces display 1–6 removal negatives, most frequently two (n = 10) or three (n = 10), with fewer showing one (n = 5) or four and more (n = 5). Removals are predominantly unidirectional (n = 13) or bidirectional (n = 10), with some orthogonal (n = 5), centripetal (n = 1), and multidirectional (n = 1) patterns. Striking platforms are morphologically varied: plain (n = 18), absent (n = 7), faceted (n = 6), with punctiform (n = 3) and cortical (n = 4) types less frequent. This variability mirrors that observed in the simple flakes and suggests differing degrees of preparation.

Macroscopic use-wear is evident on two flakes, affecting unmodified lateral cutting edges with angles between 30° and 45°. These functional zones lack associated backs or gripping surfaces, indicating expedient edge use.

#### 3.1.6 Retouched pieces.

Five complete pieces exhibit retouch removals. Three pieces are morphologically different from the rest of the flakes. They have convex dorsal surfaces, flat ventral sides, and plano-convex cross-sections. For these pieces, measurements were taken according to the morphological axis:

- Piece no. 207, made of gray jasper, measures 2.5 cm in length, 1.2 cm in width, and 0.5 cm in thickness. It is the smallest of the three and is a fragment of a retouched flake with penetrative removals. The angles range between 50° and 65°.
- Piece no. 256, made of dolomite, measures 4.4 cm in length, 2 cm in width, and 1.6 cm in thickness. It features steep unifacial removals on the dorsal side of a thick flake, which has resulted in the creation of a convex and short beak. The angles on this part of the piece range from 70° to 80°. In the distal part, the edge is rough and fine with use-wears where the angles range from 55° to 70°.
- Piece no. 8, made of black jasper, has a length of 4.5 cm, a width of 4 cm, and a thickness of 1.8 cm. The retouching consists of three long semi-steep unifacial removals on one of the lateral edges. This same edge shows macroscopic use-wears. The angles range between 40° and 70°.

Finally, one piece (3.5 x 3.2 x 0.8 cm) features a disto-lateral retouched rostrum directly prolonged by a retouched denticulated edge created by invasive semi-steep removals. A piece with similar dimensions is characterized by a small distal platform and two potentially damaged lateral edges: one rough edge with macroscopic use-wears and a cutting angle (30° to 50°), and a retouched edge (>80°).

The largest cutting tools measures 6.5 x 3.6 x 1.9 cm and is an elongated flake that has been retouched with low angle retouch that forms a small distal scraper with a simple bevel and a cutting angle between 70° and 85°. The toolkit also includes a small globular quartzite hammerstone (4.8 x 4.7 x 4.2 cm). The percussion marks are distributed around the entire periphery.

### 3.2 60–70 cm spit

#### 3.2.1 The cores.

Among the five cores from this layer, two are block cores and three are flake cores. The block cores display between two to six negatives of multidirectional and bidirectional removals. A maximum of two knapping surfaces is observed. These cores retain large cortical surfaces, suggesting that the supports were not prepared and that the knapping sequences were short. The produced flakes are short and either cortical or semi-cortical, which corresponds well to the flakes in the assemblage, and a few negatives of blades are visible.

The flake cores (n = 3) show from four to six removals on one to three knapping surfaces. These negatives are predominantly multidirectional (n = 2). These cores produced short flakes and semi-cortical flakes of various shapes. Both groups of cores also suggest the use direct freehand percussions.

#### 3.2.2 Cortical and semi-cortical flakes.

In this group (n = 14), there is a single first flake. The semi-cortical flakes (n = 13) are all short and small (<3 cm), varying in shape. Only one piece has a remnant of cortical surface in the distal part, while the others consistently exhibit a lateral or latero-distal cortical surface. These flakes show fragmented (n = 7), plain (n = 4), faceted (n = 1), and cortical (n = 1) platforms. This suggests, as within the 80–100 cm spit, that the knapping primarily opens striking platforms, followed by a phase of lateral extension of the knapping surfaces through unidirectional flaking.

#### 3.2.3 The simple flakes.

The simple flakes (n = 14) measure consistently under 3 cm in length (mean: 2.52 cm; width: 2.48 cm; thickness: 0.69 cm). Dorsal surfaces exhibit 1–6 removal negatives, most commonly two (n = 5), one (n = 3), or three (n = 3), with bidirectional (n = 6) and unidirectional (n = 3) orientations dominating. Less frequent patterns include orthogonal (n = 2), multidirectional (n = 2), and indeterminate (n = 1) removals. Striking platforms are morphologically diverse: dihedral (n = 3), punctiform (n = 3), plain (n = 3), faceted (n = 2), cortical (n = 1), and absent (n = 1), reflecting a range of preparation levels. One flake (no. 46) displays macroscopic use-wear on the proximal edge, extending distally on both sides of the cutting bevel. Edge angles range from 35° to 50°, adjacent to a possible flat fracture plane interpreted as a gripping area.

#### 3.2.4 The débordant flakes.

The *débordant* flakes are small to medium in size, ranging from 2 to 5 cm in length. Similar to the products of 80–100 cm spits, two flakes display a double overflow characterized by two opposite lateral facets and a convex dorsal surface. Six flakes exhibit lateral cortical *débordement*, two with negatives on the lateral core-edge surface and one with a plain core-edge lateral surface. On the dorsal surfaces, there are predominantly one or two unidirectional removals. There are no visible macroscopic use-wears or retouch, but a certain homogeneity in their production is evident through the type of platform, the organization of dorsal negatives, and the type of core-edge lateral surface.

#### 3.2.5 Retouched pieces.

Specimen No. 50 is a small fragment of a gray jasper flake, slightly retouched with long, abrupt, and discontinuous peripheral and unifacial removals. These removals result in a small tool with a convergent edge and a rectangular to trapezoidal cross-section, featuring cutting angles between 60° and 90°, characterized by simple bevels. One edge, slightly damaged, may have been used.

### 3.3 50–60 cm spit

#### 3.3.1 The cores.

Two cores come from small blocks of jasper, while the third appears to be on a cortical flake. All three cores exhibit a preferential exploitation surface. Two cores are designed with two opposing surfaces and feature finely prepared striking platforms, whereas the third core is characterized by unidirectional production of small flakes from a flat, cortical, but unprepared striking platform.

#### 3.3.2 Semi-cortical flakes.

The flakes are all semi-cortical (n = 7): laterally (n = 3), bilaterally (n = 1), and latero-distally (n = 3). Most of these flakes are small, not exceeding 3 cm in size. Only flake No. 163 measures over 6 cm in length with a width of 4.7 cm. It exhibits a latero-distal and cortical core-edge lateral surface. For these flakes, the striking platforms are most often fragmented (n = 4); there are also cortical (n = 2) and plain (n = 1) platforms. They have one (n = 6) or two (n = 1) unidirectional removal negatives. This suggests, as with the first two decapitation stages, that these are reduction sequences prioritizing lateral decortication of the debitage surfaces.

#### 3.3.3 The simple flakes.

The simple flakes (n = 30) are small in size (<4 cm). The average length is 2.58 cm, the average width is 2.35 cm, and the average thickness is 0.69 cm. Among these flakes of various shapes and forms, eight are short flakes. The number of negatives on the dorsal surface ranges from a one to six. Twelve flakes exhibit three negatives, while others show two (n = 5), five (n = 5), one (n = 4), and less frequently four (n = 3) or six (n = 1). These removals are most often bidirectional (n = 15) and unidirectional (n = 8), and less commonly multidirectional (n = 6) or centripetal (n = 1). This indicates a certain variability in the reduction sequence, also reflected in the morphology of the striking platforms, which can be faceted (n = 9), dihedral (n = 5), plain (n = 5), cortical (n = 4), punctiform (n = 3), or fractured (n = 4). None of these flakes show visible macroscopic use-wears.

#### 3.3.4 Elongated flakes.

The elongated flakes (n = 11) are small in size, ranging from 2.5 to 4.5 cm in length. The average lengths, widths, and thicknesses are 3.15 cm, 1.52 cm, and 0.6 cm, respectively. The reduction process on these flakes leaves between two and five removal negatives on the dorsal surface. These removal negatives are often bidirectional (n = 6) and unidirectional (n = 2), and less commonly orthogonal (n = 2) or multidirectional (n = 1). The flakes exhibit various types of striking platforms, including absent platforms (n = 2). The platforms are plain (n = 4), cortical (n = 2), punctiform (n = 2), or faceted (n = 1).

#### 3.3.5 The débordant flakes.

The *débordant* flakes (n = 8) are less than 4 cm in length. They display two types of *débordement*: bilateral (n = 2), featuring two opposing flats without cortex, and unilateral (n = 5), two of which retain remnants of cortical surface. These flakes have diverse morphologies. Specimen No. 24 displays macroscopic use-wears on the edge opposite the lateral core-edge surface, with an angle ranging from 30° to 40°. This same flake has a fully cortical striking platform.

#### 3.3.6 Retouched pieces.

Only two small flakes (<3 cm long) are retouched. Both are small jasper flakes. The first one is characterized by a short, abrupt retouch with a cutting angle between 50° and 70°, very regular along a disto-lateral edge, forming a small disto-lateral point directly extending into an unmodified sharp edge with an angle between 30° and 50°. This corresponds to an edge regularization retouch.

The second piece is characterized by abrupt retouching on both opposite lateral edges of the flake. This retouching closely resembles backing and may correspond to small modifications. This techno-functional organization corresponds to the micro-tranchet type [3, 61].

### 3.4 30–40 cm spit

#### 3.4.1 Cortical and semi-cortical flakes.

Among the cortical and semi-cortical flakes (n = 14), there are initial flakes (n = 2), semi-cortical flakes with lateral cortical surface (n = 9), and flakes with lateral-distal cortical surface (n = 3). These flakes are less than 5 cm in length, with a minimum length of 2.2 cm and a maximum of 4.4 cm. The maximum number of negatives is two (n = 2), with the majority having only one (n = 10). These removals are mostly unidirectional (n = 11). The striking platforms are mostly fractured (n = 7) and plain (n = 6) or punctiform (n = 1). No macroscopic use-wears are reported.

#### 3.4.2 The simple flakes.

The simple flakes (n = 33) are uniformly small (<4 cm), with an average length of 3.07 cm, width of 2.60 cm (max. 4.3 cm), and thickness of 0.76 cm (max. 1.5 cm). Short flakes (length < width) are present (n = 8). Dorsal surfaces exhibit 1–7 removal negatives, most commonly three (n = 12) or two (n = 10), with fewer flakes showing one (n = 5), four (n = 3), six, or seven (n = 1 each). Removal orientations are predominantly unidirectional (n = 20), with lesser representation of bidirectional (n = 7), multidirectional (n = 3), orthogonal (n = 1), and centripetal (n = 1) patterns. This diversity suggests varied reduction strategies. Striking platforms are equally variable: faceted (n = 9), dihedral (n = 5), plain (n = 5), punctiform (n = 3), and cortical (n = 3), with fractured platforms recorded on seven flakes. Macroscopic use-wear is observed on a single flake, featuring two unmodified concave edges with cutting angles below 40°, indicating potential expedient use.

#### 3.4.3 Elongated flakes.

The elongated flakes (n = 10) are mostly small, with an average length of 3.66 cm, an average width of 1.88 cm, and an average thickness of 0.62 cm. These values are well below the maximum lengths, widths, and thicknesses, which are 5.2 cm, 3.1 cm, and 6.2 cm, respectively. The dorsal surfaces show between one and four removal negatives, and several pieces retain remnants of cortical surfaces. Most commonly, there are two negatives (n = 5), less frequently three (n = 2), four (n = 2), and one dorsal negative (n = 1). These are either unidirectional (n = 6) or bidirectional (n = 4). The elongated flakes from this layer exhibit four types of striking platforms: plain (n = 5), cortical (n = 3), punctiform (n = 1), and faceted (n = 1). Specimen No. 137, covered with 80% cortical surface on its dorsal face, displays macroscopic use-wears on both the distal and lateral edges, both of which are unretouched. On the lateral edge, this wear has created a notch in the mesial section, with a cutting angle ranging from 25° to 40°.

#### 3.4.4 The débordant flakes.

*Débordant* flakes have lengths ranging from 4.5 to 2.5 cm and feature one to two unidirectional removal negatives on the dorsal face. All the striking platforms are plain, and one piece exhibits macroscopic use-wears on the *débordant* edge, suggesting the use of this abrupt lateral edge with an unretouched angle of 85°.

### 3.5 10–20 cm spit

Only six flakes were recovered from the 10–20 cm spit. First, there are two simple flakes, the longest measuring 4.5 cm in length. The width is 3.6 cm, and the thickness is 1.7 cm. Both flakes have faceted platforms. One flake (No. 3) has nine orthogonal removal negatives and is an *débordant* flake that extends to the distal surface of the *débitage* surface. The other flake has five multidirectional removal negatives and macroscopic use-wears on the latero-distal edge, along with a notch on the lateral edge. The angles of this edge with use-wears range between 30° and 40°. Next, two small flakes are semi-cortical, and one is an initial flake. The semi-cortical flakes have cortex on the distal and latero-distal parts with a unidirectional dorsal negative. Two flakes have faceted platforms, while the platform of the other flake is absent. The final piece is a short *débordant* flake with cortical edge-core lateral surface, featuring two unidirectional removals on the dorsal surface.

## 4. Lithic analysis of the SDG test-pit

### 4.1 Stratigraphic Unit 2

#### 4.1.1 Cortical and semi-cortical flakes.

Four are primary flakes and five are semi-cortical. Only one flake has a cortical distal part, while four display lateral cortical surface. These flakes are < 5 cm long. The same applies to the widths, with a minimum value of 1 cm. The flakes collectively display cortical (n = 5), faceted (n = 2) or fractured (n = 2) platforms.

#### 4.1.2 The simple flakes.

The simple flakes (n = 7) are small in size, measuring from 2.8 to 4 cm in length, with an average width of 2.1 cm, and an average thickness of 0.6 cm. These flakes exhibit cortical (n = 2), dihedral (n = 2), faceted (n = 2) platforms, and one flake with a fractured platform. The dorsal surfaces predominantly display two negative removals (n = 6), with one piece showing three negative removals. These removals are consistently unidirectional (n = 4) or bidirectional (n = 3).

#### 4.1.3 Elongated flakes.

Elongated flakes (n = 6) display length ranging from 3 to 6 cm, a width ranging from 3 cm to 2 cm, and a thickness ranging from 0.8 to 1.7 cm. These flakes display various types of platforms, including cortical (n = 2), punctiform (n = 2), dihedral, or plain (n = 1). They display between one and three negative removals, with the directions being predominantly unidirectional (n = 3), bidirectional (n = 2), and centripetal (n = 1). Three of these flakes are elongated with convergent distal edges. None of the pieces show retouch or signs of use-wear.

### 4.2 Stratigraphic Unit 3

#### 4.2.1 The cores.

**Fig 7:**
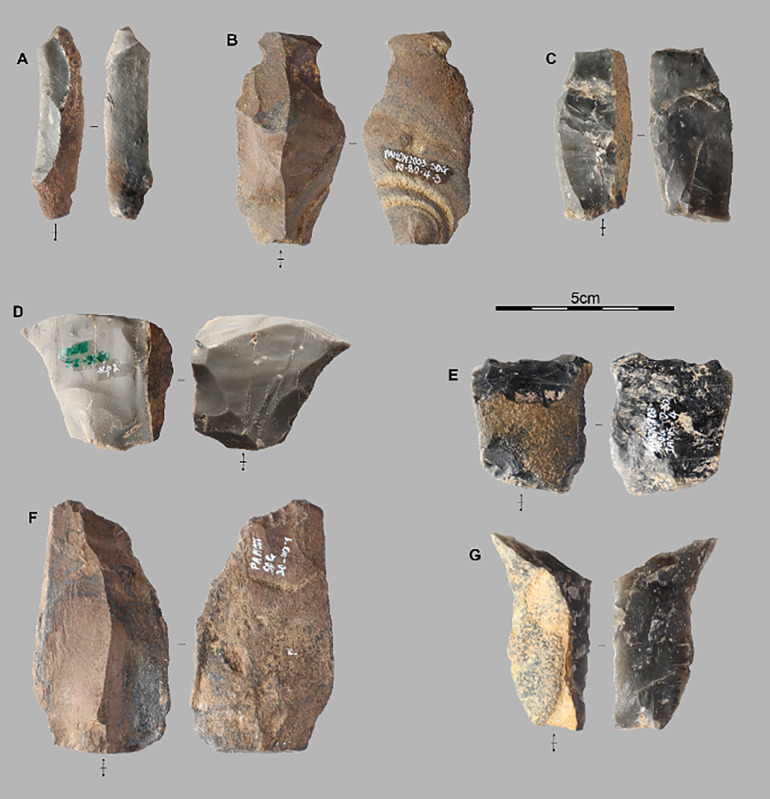
Lithic assemblages from the Pahon Cave. Examples of cores from Stratigraphic Unit 3: A is a centripetal core on a pebble, B on a block, C and D on flakes.

The core assemblage (n = 21) consists of small to medium-sized pieces (mean dimensions: 4.7 × 3.7 × 2.1 cm; Fig 7), with size ranges of 2.4–6.2 cm in length, 1.7–5.4 cm in width, and 1.2–3.3 cm in thickness. The blanks are varied, including small blocks (n = 11), flakes (n = 6), and small pebbles (n = 4), three of which are in quartz. Several cores exhibit flat natural or flaked surfaces (n = 4). Cortical coverage is frequent (n = 14), and flake cores often retain ventral or fracture surfaces (n = 3). The products obtained include cortical or semi-cortical flakes, short flakes, small blades, and, more rarely, elongated flakes. No cores are dedicated to bladelet production.

Knapping strategies include unidirectional, bidirectional, and multidirectional removals, using longitudinal, discoidal, and orthogonal approaches. Reduction sequences are generally short and unprepared, though a few cores show more structured exploitation, with two opposing flaking surfaces and prepared platforms bearing multiple impact

#### 4.2.2 Cortical and semi-cortical flakes.

**Fig 8:**
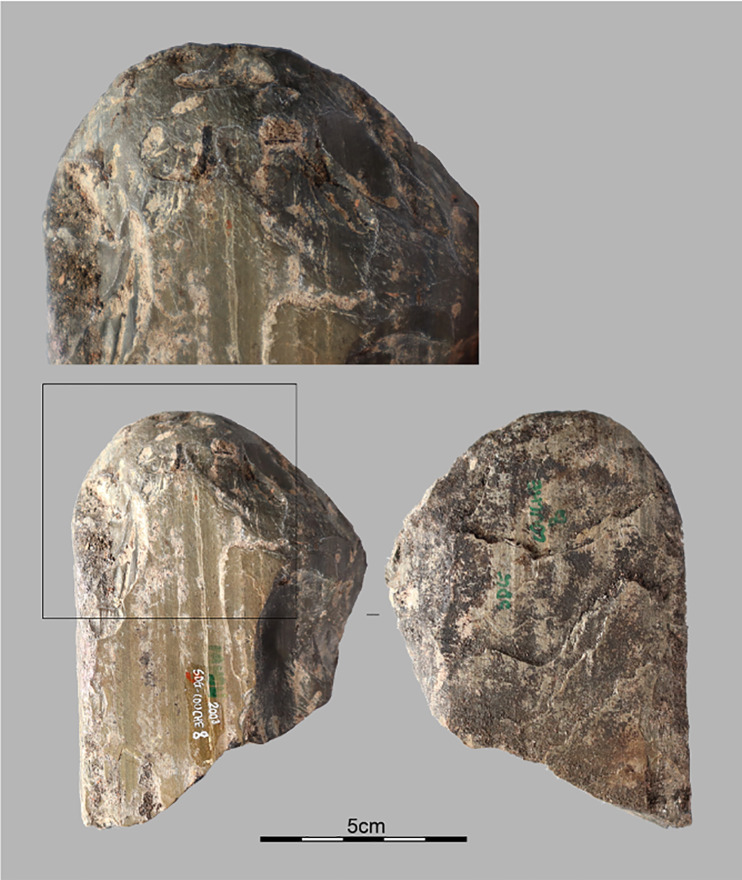
Lithic assemblages from the Pahon Cave. Flakes from Stratigraphic Unit3: A, B, and C are elongated flakes; D and F are retouched flakes; E and G are cortical and semi-cortical flakes.

Cortical and semi-cortical flakes (n = 159; Fig 8A, 8C and 8E) show considerable dimensional variability, with lengths ranging from 1.6 to 9 cm, widths from 0.8 to 4.5 cm, and thicknesses from 0.2 to 2.5 cm. Primary flakes are well represented (n = 80). Among semi-cortical flakes (n = 79), cortex is located distally (n = 23), laterally (n = 40), or proximally (n = 16). These flakes typically exhibit 1–3 unidirectional or bidirectional removals. Elongated forms are common (n = 59), often corresponding to blades (L ≥ 2W) or convergent flakes. Striking platforms are frequently absent (n = 81) or cortical (n = 41); other types include faceted (n = 15), plain (n = 10), dihedral (n = 9), and punctiform (n = 3).

Macroscopic use-wear was identified on a single primary flake. The edge in question, located on the left lateral margin of the ventral face, was thinned by a removal and used at a sharp angle (30°), contrasting with blunter adjacent angles (65°–95°). This suggests intentional use of the sharpest edge segment.

#### 4.2.3 The débordant flakes.

The *débordant* flakes (n = 66) are small (length < 5 cm; min. 1.8 cm), with widths ranging from 1 to 3.3 cm and thicknesses below 2 cm. Some exhibit core-edge surfaces on both margins (n = 7), while others retain cortex on the *débordant* edge (n = 29) or display 1–4 removal negatives on these edges (n = 11). Dorsal surfaces show 1–5 negatives with varied orientations. Striking platforms are diverse: absent (n = 25), faceted (n = 13), cortical (n = 11), plain (n = 8), dihedral (n = 6), and punctiform (n = 3), indicating varied core configurations for *débordant* flake production.

Macroscopic use-wear is observed on seven unretouched flakes. The worn edges consistently exhibit angles <40°, opposed to the core-edge surface, suggesting expedient use. Morphologically, these flakes (elongated, short, or convergent) align with the broader assemblage, and some cores match their characteristics.

#### 4.2.4 Simple flakes.

Simple flakes (n = 125) are characterized by a mean of of 2.8 cm in length, 2.5 cm in width, and 0.7 cm in thickness. There are 30 short flakes. The dorsal negatives range from 1 to 6, with the most common being two negatives (n = 44), followed by one negative (n = 38) and three negatives (n = 26). The majority of these removal negatives are unidirectional (n = 74) and bidirectional (n = 30), though there are also multidirectional (n = 16) and centripetal (n = 5) negatives. A variety of striking platforms is observed: cortical (n = 29), plain (n = 26), faceted (n = 24), absent (n = 22), dihedral (n = 13), and punctiform (n = 11).

Within this group, seven flakes show macroscopic use-wears, with four located on the distal part and three on the lateral part and systematically on unmodified cutting edge with angles <60°. These flakes exhibit a variety of shapes and sizes, suggesting a lack of standardization in the unmodified flakes used except that all present thin cutting edge.

#### 4.2.5 Elongated flakes.

The elongated flakes (n = 97) exhibit various shapes and sized including convergent flakes (n = 13), and unlike other spits, there is an abundance of blades (n = 27). The average dimensions, including the blades, are 3.6 cm in length, 1.6 cm in width, and 0.7 cm in thickness. These pieces result from diverse knapping methods, evident in the various directions of removals: dorsal removals vary from 2 to 5 scars, with unidirectional (n = 74), bidirectional (n = 16), or multidirectional (n = 7) orientation. The striking platforms on these flakes are plain (n = 37), cortical (n = 21), or faceted (n = 20). Less commonly, they are fractured (n = 11), dihedral (n = 5), or punctiform (n = 3).

Nine elongated flakes display macroscopic use-wears on the two opposing edges (n = 7) or in a latero-distal position (n = 2) with angles varying between 45° and 25°.

#### 4.2.6 Retouched pieces.

**Fig 9:**
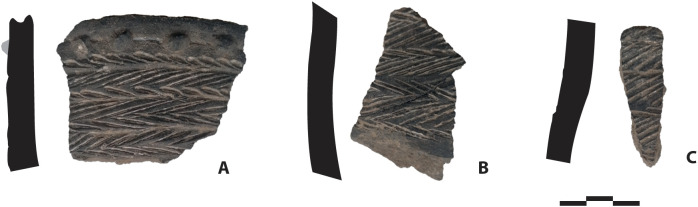
Fragment of a polished axe. Polished adze fragment from Stratigraphic Unit 3 dated between 6,604 and 5,660 cal BP.

We have ten pieces with intended edge modification by retouch, including a fragment of a polished axe Fig 9. The polished axe is fractured and appears to have been shaped from a slab, though the raw material has not been determined yet. On edge is convex and the opposite one is rectilinear. The tool is only partially polished with visible removals on one of its faces. These removals have thinned one lateral edge, forming the cutting edge. This edge has an angle ranging from 65° to 80°. In contrast, the right lateral edge has angles between 80° and 120°, making it more robust.

Other retouched flakes exhibit diverse shapes. The mean dimensions are 4.3 cm in length, 3.1 cm in width, and 1.2 cm in thickness. Among the retouched flakes, there are three blades, four *débordant* flakes, one simple flake, and one short flake and are all integrated in the diversity of the *débitage* described above.

The retouch removals are located on the lateral edges (n = 4), on the disto-lateral areas (n = 3), and on the proximal and distal ends (one flake each). The inclinaison of the retouch range from low-angle to abrupt and consist of short removals only. They do not alter the morphology of the pieces as would be seen during rejuvenation [56]. They appear to have been intended to regularize the cutting edge. The angles of the retouches range between 35° and 85°.

### 4.3 Stratigraphic Unit 4

#### 4.3.1 Cortical and semi-cortical flakes.

Among the cortical and semi-cortical flakes (n = 22), there are 12 primary flakes with a maximum length ranging from 5 to 3 cm. Then, there are the semi-cortical flakes (n = 10), which have a maximum length of 6 cm and a minimum length also less than 3 cm. They feature cortex on the lateral part (n = 5) and on the distal part (n = 5). The flakes are small to medium in size. Most of them have absent platforms (n = 12), cortical platforms (n = 5), plain platforms (n = 2), and faceted platforms (n = 2).

#### 4.3.2 The simple flakes.

The simple flakes group (n = 10) includes four short flakes (n = 4) and a length mean <4 cm. These flakes exhibit several types of striking platforms: plain (n = 3), cortical (n = 5), and dihedral (n = 2). The number of removal negatives ranges from 1 to 7 but the most common are two removal negatives (n = 4) and three (n = 3). None of these flakes show retouch or use-wears.

#### 4.3.3 Elongated flakes.

Elongated flakes (n = 6) have with a length varying from 3 to 8 cm, the width comprised between 1 and 4 cm and thickness between 0.4 and 2 cm. These elongated flakes also show a variety of striking platforms: plain (n = 2), dihedral (n = 2), faceted (n = 1), and cortical (n = 1). The number of removal negatives ranges from one to six, including three (n = 2), one (n = 2), six (n = 1), and four (n = 1). These removals are bidirectional (n = 3), unidirectional (n = 2), and centripetal (n = 1). The longest elongated flake shows macroscopic use-wears on both opposing edges, with angles varying from 30° to 45°.

#### 4.3.4 Retouched pieces.

Retouched pieces are all flakes with lateral retouch, each under 6 cm in length and a maximum width of 3 cm. The thickness varies from 0.6 to 1.8 cm. One flake displays an abrupt retouch along the lateral edge, with an angle ranging between 45° and 35°. The other two flakes display a retouched area extended from the mesial to the distal part. The angle of this retouch ranges between 45° and 30°.

### 4.4 Stratigraphic Unit 5

#### 4.4.1 The cores.

The assemblage consists of two cores, < 5 cm; < 3 cm in width and <2 cm in thickness. They exhibit between 2 and 5 unidirectional removals. These two cores are small pebbles corresponding to simple, cortical, and semi-cortical flakes.

#### 4.4.2 Cortical and semi-cortical flakes.

Cortical flakes (n = 22) range in size from small to medium. Among them, 9 are primary flakes and for semi-cortical flakes, the cortical area is mostly lateral (n = 9), distal (n = 3), and proximo-lateral (n = 2). The semi-cortical flakes have between one and four removals on the dorsal surface, most often unidirectional, with only two flakes showing bidirectional removals. The striking platforms of these flakes are often cortical (n = 11) or fractured (n = 8). Additionally, three flakes have faceted platforms, and one has a punctiform platform. None of these unmodified flakes appear to have been used or retouched.

#### 4.4.3 The débordant flakes.

*Débordant* flakes vary in length from 3 to 5 cm, and in width from 2 to 5 cm, and in thickness from 0.5 to 1.5 cm. The group comprises cortical (n = 9) and non-cortical (n = 3) *débordant* flakes. Additionally, two flakes show a core-edge surface on both sides (n = 2). On their dorsal surfaces, there are between one and four negative removals, mostly unidirectional (n = 10), with a smaller number being multidirectional (n = 2). The flakes feature seven cortical (=7), faceted (n = 4) or plain (n = 1) platforms. Among these flakes, one piece shows macroscopic use-wears. These use-wears are visible along the entire left lateral edge, extending to the distal part, with angles ranging from 40° to 25°.

#### 4.4.4 The simple flakes.

These are small flakes (n = 10) that do not exceed 3 cm in length, with an average length of 2.7 cm. The average thickness and width are 0.7 cm and 2.2 cm, respectively. As for the maximum and minimum values, they are 3.4 cm and 2 cm for length, 3.8 cm and 1 cm for width, and 1 cm and 0.4 cm for thickness. All these flakes display two directions of removals on the negatives visible on the dorsal face. The majority have unidirectional negatives (n = 8), while two flakes are bidirectional. The number of negatives ranges from one to four.

#### 4.4.5 The elongated flakes.

There are 26 elongated flakes, ranging from small to medium-sized. The averages for the entire set of flakes are 4.5 cm in length, 2.7 cm in width, and 0.8 cm in thickness and the group presents considerable metric variability. The majority of these elongated flakes do not correspond to the cores from the SU5, as they are significantly larger than these cores. They display between one and six dorsal negatives. The most common is three removals (n = 11), followed by two removals (n = 6) and one (n = 3). The removals are unidirectional (n = 15), bidirectional (n = 7), and centripetal (n = 4). The butts exhibit varied morphologies: faceted (n = 14), cortical (n = 8), or plain (n = 4). Three pieces show macroscopic use-wears. These use-wears are visible on the disto-lateral part for two flakes and the angles range between 30° and 45°. Two elongated flakes present macroscopic use-wears on both lateral opposite edges with angles values ranging from 40° to 65°.

## 5. Analysis of faunal remains

During the excavations, faunal remains were collected by dry sieving on a 4 mm mesh, allowing the retrieval of numerous small bones. The material was identified by comparison with the reference collections of the Royal Museum for Central Africa, Tervuren.

The faunal material consists of 1045 remains of which 950 were determined. Table 3 presents an overview of the archaeozoological remains per stratigraphic unit.

**Table 3:** Overview of the faunal remains identified in the different stratigraphic units of the test pits SDG and TOB. The figures represent number of identified specimens (NISPs).

	SDG							TOB						
Stratigraphic unit	#1	#2	#3	#3-4	#4	#5	total	0-20 cm	30-40 cm	40-60 cm	70-80 cm	80-100 cm	total	grand total
giant African snail (*Achatina* sp.)	4	18	5	–	–	1	28	3	1	1	3	5	13	41
freshwater crab (Potamonautidae indet.)	–	4	–	–	–	–	4	–	–	–	–	1	1	5
frog or toad (Anura indet.)	–	–	–	–	–	–	–	–	–	1	–	–	1	1
owl (Strigiformes indet.)	–	–	–	–	–	–	–	–	–	1	–	–	1	1
unidentified bird	–	1	–	–	–	–	1	–	–	–	4	–	4	5
fruit bat (*Rousettus* sp.)	–	–	19	10	–	1	30	–	–	–	–	–	–	30
giant roundleaf bat (*Macronycteris gigas*)	–	2	5		–	1	8	62	8	138	234	9	451	459
unidentified large bats	–	8	21	16	–	5	50	68	16	96	137	25	342	392
brush-tailed porcupine (*Atherurus africanus*)	1	–	–	–	–	–	1	–	–	–	–	–	–	1
unidentified carnivore, *Atilax paludinosus* size	–	2	–	1	–	–	3	–	–	–	–	1	1	4
bushpig (*Potamochoerus porcus*)	–	1	–	–	–	–	1	1	–	–	–	1	2	3
unidentified suid (Suidae indet.)	–	–	–	–	–	–	–	–	–	–	–	1	1	1
water chevrotain (*Hyemoschus aquaticus*)	–	1	–	–	–	–	1	–	–	–	–	–	–	1
duiker (*Cephalophus* sp.)	–	2	1	1	–	–	4	1	–	–	–	1	2	6
unidentified mammal	6	20	2	13	7	3	51	12	1	6	6	19	44	95
total	11	59	53	41	7	11	182	147	26	243	384	63	863	1045

A total of 41 fragments have been found of large gastropods belonging to the genus *Achatina*. Identification to species level was not possible as the original colours were lost due to taphonomic processes. These giant African snails are typically harvested in forested areas of Africa and form an important source of animal protein [62]. The only other invertebrate remains found in Pahon Cave are pincers of small crabs, that must have lived in the small river that runs through the cave, as still is the case today. We attribute the remains to the family of the Potamonautidae, the only family of freshwater crabs living in Gabon (C. d’Udekem d’Acoz, pers. comm.). An isolated tibiofibula, that on an osteological basis could belong to either a frog or toad, is another indication for an animal associated with aquatic environments. Two frog species have been discovered recently in the cave during a systematic zoological survey [63], showing that these creatures occurred here naturally. Bird remains include a distal tibiotarsus of a medium-sized owl, and four eggshell fragments that could not be brought to species.

The great majority of the faunal remains is from large bats among which two taxa could be identified. The most abundant species are the giant roundleaf bat (*Macronycteris gigas*) which is still living in the cave today. Fruit bats of the genus *Rousettus* account for only 6% of all identified bat remains. All major skeletal elements (skull, mandible, long bones) of both taxa could be identified, whereas less diagnostic pieces such as vertebrae and shaft fragments remained unidentified. The state of preservation is highly variable, going from unmodified, rather intact bone of light brown colour to more or less fragmented pieces with traces of fire that gave the bones a black, grey or whitish colour. Both *Rousettus* and giant roundleaf bat inhabit caves, and often both taxa co-occur [64]. The latter species is considered a delicacy in Gabon and Cameroun where they are often captured in large quantities by smoking caves, resulting in suffocation of the animals [64]. Because of its weight, *Rousettus* is also sought after for food. In the taphonomic discussion below, we will discuss whether the bat remains from the Pahon **C**ave should be seen as human food waste or rather as remains of carcasses of animals that died a natural death on site.

Among the larger mammal remains is a single bone, a fibula, of brush-tailed porcupine (*Atherurus africanus*). This rodent is a typical forest dwelling species that is often found in crevices or hollow trees near small water courses [65]. Humans are the main predator of this species that is often captured with snares set on their trails [66]. It was not possible to attribute an exact species to the thoracic vertebra, the humerus shaft, the unfused proximal femur and the pelvis fragment of a medium-sized carnivore. It is comparable in size to marsh mongoose (*Atilax paludinosus*). Mongoose, but also other medium-sized carnivores occurring in the region, such as genets, are edible and found on bushmeat markets in Gabon [67]. One of the four suid remains, a vertebra fragment, could not be identified beyond family level. The three other bones, a proximal fibula, a fourth metacarpal and a fourth metatarsal, were identifiable as bushpig (*Potamochoerus porcus*). This is a gregarious species typically living in rainforest or gallery forest that is nowadays much sought after in Gabon because of its high meat yield (average weight 80 kg) [68]. A distal metatarsal is the only indication for the presence of water chevrotain (*Hyemoschus aquaticus*). This terrestrial species typically lives in the rainforest, and is usually found near water in which it takes refuge when in danger [69]. Because of its nocturnal lifestyle, snaring may have been the most effective capture method in the past.

Six antelope remain, a jugal bone, a thoracic vertebra, and shaft fragments of a humerus, a femur, a metacarpal and a metatarsal, pertain to a medium-sized duiker (*Cephalophus* sp.). Animals of this size category are sometimes referred to as red duikers, which then include *Cephalophus callipygus*, *C. dorsalis*, *C. leucogaster*, and *C. nigrifrons*, as opposed to the larger yellow-backed duikers (*C. silvicultor*) and the smaller blue duiker (*Philantomba monticola*) [70]. The exact species of red duiker could not be identified, but they are all typical of wooded areas and are nowadays an important source of bush meat.

## 6. Interpretation

### 6.1 Synthesis of the technological data from TOB and SDG

A total of 1,131 lithic artifacts were recorded from the two excavation units, TOB (n = 388) and SDG (n = 773), including *débris*, fragments, and natural pieces that were not analyzed. The total number of studied artifacts is 985.The detailed analysis provided by the samples and the stratigraphic unit reveals a form of stability, as the lithic assemblages show consistent knapping objectives aimed at producing the same types of products over a span of 5,000 years.

The material from the SDG area is nearly double that of TOB, likely because the volume excavated is twice as large. Debitage products are the most abundant in both units, comprising 88.3% of the assemblage. The most common types are cortical and semi-cortical flakes, representing 28.5% (n = 281), simple flakes at 27.9% (n = 275), and elongated flakes at 19.7% (n = 194). *Débordant* flakes are less frequent, accounting for 12.1% (n = 120). Cores (n = 47) make up 4.7% of the material. Additionally, there are 41 flakes with macroscopic use-wears (4.2%) and 24 retouched pieces (2.4%). The rarest objects include a hammerstone and a fragment of a polished axe, with the latter contributing an early date to the development of stone polishing in Atlantic Central Africa.

### 6.2 Core exploitation

Pahon Cave’s cores often exhibit similar characteristics Table 4. Regarding block-blank cores (BBC) and flake-blank cores (FBC), they are the most frequent, while pebble-blank cores (PBC) are less common. BBC and FBC cores are more prevalent at the TOB excavation site, whereas FBC cores dominate at SDG. Both BBC and FBC are equally represented overall. Various flaking methods are observed among these cores, with bidirectional being the most common, followed by unidirectional. Orthogonal and centripetal flaking methods are the least frequent.

**Table 4:** Comparison of techno-typological attributes of cores from TOB and SDG trenches.

Patterns		TOB (23 cores)	SDG (24 cores)	Total
**Core type**	BBC	12	6	18
	FBC	7	12	19
	PBC	4	6	10
**Removals number**	1–5	13	8	21
	6–11	10	14	24
	Over 12	0	2	2
**Number of surfaces**	1	2	0	2
	2	15	7	22
	3	6	12	18
	4	0	4	4
	5	0	1	1
**Number of flaking surfaces**	1	5	1	6
	2	11	10	21
	3	7	12	19
	5	0	1	1

These cores often retain residual cortical surfaces. For non-cortical cores, up to 20 removal negatives are observed. The most common sequence involves between six and eleven removals, while sequences with twelve to twenty removals—representing the longest reduction sequences—are less frequent. At the TOB site, cores typically display sequences with between one and five removal negatives, indicating shorter operational sequences.

Most cores feature between two and three flaking surfaces, with cores having four or just one face being rarer. In terms of surface exploitation, the cores generally show between one and five exploited faces, though two or three flaking surfaces are the most common.

### 6.3 Patterns of the flaking strategies

Debitage products account for more than 85% of the Pahon Cave assemblage Table 5 and are characterized by a predominance of bidirectional (n = 253) and unidirectional (n = 121) dorsal scars. This indicates predominantly longitudinal unidirectional to bidirectional flaking, which aligns with the significant representation of elongated flakes at Pahon. Additionally, the dominance of specific dorsal configurations suggests repeated knapping patterns despite variability of striking platform types.

**Table 5:** Comparison of techno-typological attributes of flakes from TOB and SDG trenches.

Pattern		TOB	SDG	Total
**Orientation of removals**	Unidirectional	69	52	121
	Bidirectional	54	199	253
	Multidirectional	0	25	25
	Centripetal	4	11	15
	Orthogonal	8	0	8
	Undetermined	6	0	6
**Scars number**	0	2	0	2
	1	24	63	87
	2	51	117	168
	3	47	69	116
	4	14	23	37
	5	9	7	16
	6	6	6	12
	7	1	2	3
**Platform types**	Plain	42	74	116
	Cortical	22	73	95
	Dihedral	21	25	46
	Punctiform	4	7	11
	Faceted	31	62	93
	Absent	24	30	50

No cores in the analyzed assemblage show clear blade removal scars, meaning there are no cores that can be directly associated with the elongated blade-like flakes. Similarly, there are no specifically laminar or lamellar cores within SU3, where the highest number of bladelets was found. This techno-typological inconsistency raises questions about the production location of these elongated products and where they were discarded, or about the knapping phase during which these elongated products were created. For instance, it is possible that the cores were fully exploited or knapped in another part of the cave. Another hypothesis is that these elongated flakes—sometimes with convergent edges—were produced elsewhere and imported into the cave for specific activities. This idea is supported by the prominence and visibility of macroscopic use-wear marks on unmodified acute edged flakes, even though such wear marks are less frequent on blade-like and bladelet-like flakes.

The abundance of cortical flakes may indicate the presence of an extensive exploitation of initial blank at the site. There are initial removal flakes and many semi-cortical flakes. Both at the TOB and SDG trenches, these flakes exhibit a diverse morpho-technological range with various types of platforms. While cortical platforms are the most common, faceted and dihedral platforms are also present, suggesting platform preparation might have occurred from the earliest phases of core exploitation. Semi-cortical flakes typically display one or two unidirectional or bidirectional removals, and some are elongated and sometimes plunged. A minority of these flakes exhibit macroscopic use-wears. Finally, the simple and *débordant* flakes vary widely in shape and size. Simple flakes are the thinnest of all, usually displaying fine cutting edges on both lateral and distal edges.

### 6.4 Retouched pieces and macroscopic use-wear

**Fig 10:**
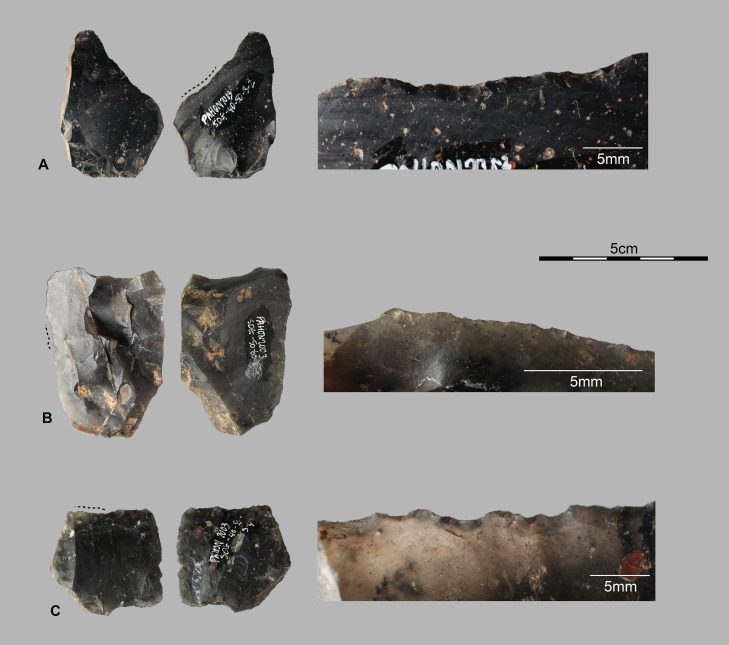
Tools with macro wear marks. Examples of macroscopic use-wears on unretouched flakes from TOB trench (Stratigraphic Unit 3). A and B present lateral cutting edge uses and C is distal edge use.

Among damaged tools and retouched pieces, no difference could be observed through the sequence. Among the artifacts exhibiting macroscopic use-wear and those with retouch Fig 10, almost all categories of flakes are represented, although simple and *débordant* flakes are the most common, with elongated flakes being rarer. Notably, convergent, cortical, and semi-cortical flakes display only macroscopic use-wears, with no evidence of intentional modification by retouch. In contrast, simple flakes, *débordant* flakes, and blades show both retouch and macroscopic use-wear.

The Pahon assemblage does not exhibit the production of characteristic or “typical” tools with the exception of six borers whose tip is formed by a natural break combined with a notch, found in both test pits.

Instead of defined tool types, the knappers’ focus appears to be on unmodified cutting edges, most often characterized by a straight, thin, double-beveled edge with angle values comprised between 60° and 30°. Some pieces could be classified as scrapers or borers, based on the location of the retouch, but no specific flake type is consistently associated with a particular retouch pattern. This suggests the absence of standardized production processes or conceptual frameworks linking specific tool types to particular retouch.

A detailed use-wear analysis would have complemented this study, potentially providing insights into specific tool movements [71] or possible uses on plant materials [57,72]. Additionally, while these macroscopic use-wears Fig 10 could be taphonomic or post-depositional in nature [73], the excellent preservation of the material and finely stratified deposits at Pahon support the interpretation that these are genuine use-wear marks. The high prevalence of artifacts with macroscopic use-wear marks on thin unmodified cutting edges is a defining characteristic of the material from Pahon Cave across all studied levels, dated between 2,724 and 7,571 cal BP.

As noted earlier, this “use retouch,” which is sometimes difficult to classify as intentional modification or wear, appears within an assemblage of small flakes (<5 cm), yet not strictly microlithic (<3 cm). The industry is centered on flakes produced through non-standardized, barely prepared, and highly diverse knapping methods. The preferred cutting edges are consistently thin and straight, with cutting angles typically less than 60°. If there was indeed an intentional pursuit of thin, acute unmodified cutting edges for as-yet-unknown activities, it would logically result in fewer retouched pieces.

A similar pattern has been observed at the neighboring Youmbidi Cave site, suggesting a possible baseline for describing lithic production sequences of the Later Stone Age (LSA) in Holocene Middle Ogooué area. It is therefore conceivable that human populations living within the forest might have chosen to utilize plant resources for tool production, as suggested at certain tropical and equatorial Asian sites [57,72,74]. Plant materials—leaves, roots, bark, or wood—can all serve various functions. S. Bahuchet documented several plant-based tools and equipment that are particularly important in the toolkits of contemporary forager groups [4]. These ephemeral objects, made from materials such as leaves or bark, are often used for single tasks before being discarded, much like their leaf-constructed huts. He adds that while these tools may seem simple, they represent the culmination of technical evolution, where “evolution” denotes change over time.

Considering the possible activities within the cave, we have ruled out butchery. Although we found a significant number of faunal remains, most are not anthropogenic. Among those that are, the majority consist of giant snails, which do not necessarily require lithic tools for preparation. For the consumption of small and medium-sized mammals, only a detailed use-wear analysis could determine whether lithic tools were employed.

### 6.5 Cultural affinities of Pahon Cave lithic remains

The first publication of the archaeological material from Pahon [16] proposed the association of the site with the Tshitolian [75,76,61], which at the time prevailed in the descriptions of the regional Later Stone Age (LSA). This first affiliation was based on chronological criteria.

The Tshitolian is a cultural facies established by Henri Breuil in 1943 [77] and later refined by J.D. Clark [78], that was defined as follows: “*Thus, the Tshitolian appears, in its initial definition, as a late Epilevalloisian industry retaining characteristic tools from the Sangoan (or ‘Tumbian’) forest cycle, while incorporating a wide variety of arrowheads, often crudely made. It can therefore be described as a forest-tradition civilization utilizing bows and arrows*” [79]. Among its typical tools are small adzes, as well as picks and gouges [80]. None of these criteria have been observed at Pahon Cave, suggesting cultural affinities other than strictly Tshitolian and raising questions about the techno-cultural diversity of the LSA in Atlantic Central Africa during Holocene [81].

While the Later Stone Age chrono-cultural phase is mainly reported in Central African sites dating to the Holocene, evidence indicates it existed as early as the Late Pleistocene, over 40,000 BP [43,48]. Contemporaneous Central African sites show a technical diversity within the Middle to Late Holocene. Because of the absence of lithic hallmarks such as arrow-heads or microliths, Pahon Cave may stand out for its seemingly “simple” flake production assemblages compared to other regional industries that persists through the entire sequence without any visible abrupt changes. Despite some artifacts of small size, the industry of Pahon Cave cannot be described as a microlithic industry. For example, Shum Laka rock shelter, Cameroon, delivered a persisting microlithic quartz industry during the Middle Holocene, dated between 6,000 and 7,000 BP, with some polished tools. Between 6000 and 3000 BP, there is a shift toward fewer microliths and more macrolithic tools, including bifacial tools, a trend continuing between around 4,000 and 3,000 BP [34,82,83].

Sites in the Ngounié region of Gabon, such as Mavanga, feature small tools, including tanged bifacial pieces [84]. The Lac Noir site near Ndendé (dated between 6,450 and 4,990 BP) and Camp de Malheur (with a microlithic industry on a variety of raw materials and including flakes, cores, scrapers, and denticulated tools) have been also attributed to the final phase of the Tshitolian [16].

Stone polishing appeared more clearly between 6,000 and 4,900 cal BP in association with bifacial tools and pottery at Shum Laka [1, 35]. This technique is part of a suite of innovations at the site (macrolithic tools, increasingly specialized gathering, ceramics), suggesting significant societal transformations and the possible emergence of horticultural practices. The presence of a similar artifact at Pahon could indicate analogous processes; however, this single find remains isolated and lacks corroboration from the neighboring Youmbidi Cave, currently under excavation [20]. Additionally, the polished axe fragment from ES3 in the SDG trench is associated with lithic material, but no pottery sherds were found at this level or in other trenches (TOB and SDG), except on the surface. However, another early occurrence is reported between 7,370 and 6,570 cal BP at Ntadi Yomba, Republic of Congo [1].

### 6.6 Interpretation of archaeozoological remains

#### 6.6.1 Taphonomy.

Cave deposits offer a suitable environment for the preservation of faunal remains. Subsurface weathering is tempered as the animal remains suffer less from alternating cycles of temperature or humidity fluctuations, compared to an open-air environment. In addition, clastic sediments from the cave roof and walls ensure a relatively rapid burial of the animal bones, limiting surface exposure. Caves also have the advantage that, except for the entrance, plant growth is absent which makes the soil less acidic compared to open air sites, certainly in a forest environment. Challenging when dealing with fauna remains derived from caves, is finding out which taphonomic agents were responsible for their deposit [85–87]. Indeed, not only humans inhabiting caves or rock shelters can leave animal bones behind. Leopards (*Panthera pardus*) and hyenas can also leave left overs of their prey in caves, and porcupine (*Hystrix* spp.) can bring bones to caves to chew on. Of these species, only leopards occur in the central African rainforest, and if present their deposited bones should bear typical gnawing and digestion traces [85]. Animal remains found in caves can also derive from sick or wounded animals that sought refuge and died on the spot, or they can come from cave dwelling species (snails, bats,..) that died there naturally. Taking into account the skeletal elements present, the traces visible on the remains, the stratigraphic context and the ecology of the various species, it can be tried to distinguish the different taphonomic groups [*sensu* Gautier, 60] within a faunal assemblage. It appears that the material from Pahon Cave contains human consumption refuse, but that the majority of the remains should be considered as so-called penecontemporaneous intrusives, i.e., animals that were not intentionally brought to the site by humans.

**Fig 11:**
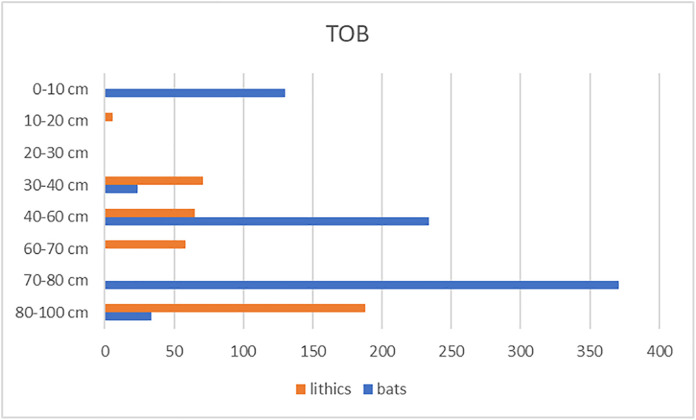
Correlation between archeozoological and lithic remains. Abundance of bat remains and lithic material within the different stratigraphic spits (fragment counts).

The remains of several species found in Pahon Cave, can be considered of anthropogenic origin, namely those of giant snail, brush-tailed porcupine, medium-sized carnivore, bushpig, the unidentified suid, water chevrotain and duiker. These are animals that are typical food species, also used nowadays, that in addition are not cave dwelling. The pincer remains of freshwater crab and the bone of a frog or toad are probably from animals that crawled into the cave themselves and that died there naturally. The bone of an unidentified owl likely is derived from an animal that roosted and died in the cave. The bird egg fragments cannot be identified, but are probably also from a species that nested near the cave entrance. No less than 93% of all identified faunal remains is from large bats that typically inhabit caves. Fruit bats of the genus *Rousettus* are only found in trench SDG in which giant roundleaf bat (*Macronycteris gigas*) is also represented albeit in smaller numbers. In trench TOB, somewhat deeper in the cave, the giant roundleaf bat is the sole bat species. Although it is known that large bats can be captured for food, usually with the aid of nets, we believe that the remains found in Pahon Cave represent mainly, if not exclusively, animals that died naturally. Interspecific associations between *Rousettus* and Hipposideridae occur frequently [64], and in this case it is striking that *Rousettus* is the only taxon found in the trench that is closest to the entrance of the cave. This is in accordance with the ecological preference of this fruit bat. The giant roundleaf bat occupies darker parts, often with very low ceilings, and forms large swarms [64]. The spatial distribution within the cave of the two bat taxa is a first indication that the bone remains represent a natural death assemblage. In the case of trench TOB, there are sufficient remains to verify the vertical distribution of the bat bones and to compare their abundance to that of the lithic material Fig 11. It appears that they are extremely abundant in the top level and in the 70–80 cm spit where lithics are absent. Also, in the level 40–60 cm they are well represented, albeit that those levels also contain many lithics. In the lowermost level, where lithics are most numerous in the sequence, bats also co-occur. We are not sure the bats occupied the cave during periods of human presence as they usually do not support prolonged disturbance. It is more likely that there was an alternation of occupancy by bats and humans. During the occupation by bats, feces and carcasses of animals that died naturally on the spot will have accumulated under the roosting places and may have been dispersed layer on by natural or anthropogenic activities.

The skeletal element representation shows that all bones are present, including fragile remains. There is no evidence for human gnaw or bite marks that could be expected if the animals would have been consumed. Several bones show traces of burning, from rather lightly charred elements with black colour to elements that have been exposed to very high temperatures judging from the white to blueish colours and the brittle nature. However, rather than seeing this as an indication for the roasting of bats, we consider this as undeliberate burning when bat carcasses accidently ended up in hearths. Roasting of bats would in any case not result in bones that were entirely burnt, including the meaty parts. In the top layer, 39% of the bat bones is burnt, in the level 30–40 cm it is 31%, in 40–60 cm 11% and in 70–80 and 80–100 only 1% and 6% respectively.

Without all these bats and the other non-anthropic remains mentioned above, the number of bones that can be assumed to be brought in by humans is limited to a mere 6% of the total identified assemblage. This low number obviously limits the possibilities of interpretation in terms of paleoeconomy.

#### 6.6.2 Animal exploitation.

The few anthropogenic remains from the faunal assemblage show that subsistence, as far as animal food is concerned, was based on the harvesting of giant snails and on the capture of medium-sized mammals. We believe that, if large bat consumption occurred at all, this must have been very occasional and it certainly did not contribute significantly to the diet. In terms of meat yield, the major food animals were, in increasing importance of weight, the brush-tailed porcupine (2–3.5 kg), medium-sized carnivores, water chevrotain (7–15 kg), duikers (20 kg) and finally bushpig (80 kg). Traditionally, traps were made of plant materials and these were efficient for the capture of all mammals encountered at Pahon cave, with the exception of bushpig that would pull down the snares [70]. Spear hunting would be efficient for the capture of bushpig, but less successful for the red duikers that are more difficult to find as they live singly or in pairs. All identifiable animal species are typical of a forested environment. The absolute number of remains are too low to verify if any shift occurred through time that could be indicative of an environmental change.

## 7. Conclusion

The Pahon Cave, excavated over 20 years ago, presents well-preserved lithic and faunal material dated between 2,724 and 7,571 cal BP. This sequence, found within the first meter of sediment, corresponds to part of the Middle to Late Holocene. It is therefore contemporaneous with significant climatic and cultural events in Atlantic Central Africa, notably the end of the “African Humid Period,” which likely contributed to the expansion of tropical forests [88], the early emergence of pottery and new subsistence strategies [37,41] and Bantu expansion [11,89].

Material from the two excavation trenches, had previously been published in a preliminary study as Tshitolian Later Stone Age [16], typically associated with various small to microlithic shaped tools. Our study, based on a technological analysis, reveals a complete—though relatively simple—lithic operational sequence, characterized by unretouched, low-standardization stone flakes, often used as unmodified tools. This is a lithic industry that does not conform to the classical typology and instead reinforces the hypothesis of the diversity of Holocene lithic technical behaviors in Central Africa [1]. Contrary to conventional views that associate the Holocene with complex and standardized production schemes, the industries from the Pahon Cave exhibit highly flexible technical systems, marked by variability and simplicity in both application and knapping know-how. A certain number of these flakes show macroscopic use-wear while a few pieces present intentional edge modification through retouch but no distinctive tool types could be identified. The technological features, raw material exploitation and metrical patterns are homogeneous through the entire sequence suggesting stable lithic technical practices through the Middle and Later Holocene.

The neighboring Youmbidi Cave has yielded similar cryptocrystalline materials, predominantly jasper, with limited use of quartz [20]. However, the discovery of a partially polished axe fragment within this assemblage raises questions about the range of tools produced or brought into the cave.

Several faunal remains suggest limited on-site consumption, as most of the remains are not directly linked to human activity (e.g., natural deaths of bats, toads, and birds). The primary food sources appear to have been giant snails and small mammals, which might have been hunted or trapped. In addition, we observe continuity in stone tool manufacture throughout the Pahon Cave sequence, despite the environmental and cultural changes occurring in Atlantic Central Africa during this time period.

## Supporting information

S1 Data Database of lithic material from Pahon Cave during analysis. (XLSX)
