# Correction: Pahon Cave, Gabon: New insights into the Later Stone Age in the African rainforest

**DOI:** 10.1371/journal.pone.0349779

**Published:** 2026-05-22

**Authors:** Marie-Josée Angue Zogo, Isis Mesfin, Geoffroy de Saulieu, Wim Van Neer, Els Cornelissen, David Pleurdeau, Richard Oslisly

Fig 1 was uploaded incorrectly. Please see the correct Figs 1 here.

The captions for Figs 4 to 9 were switched incorrectly. The caption for Fig 4 should be for Fig 9, the caption for Fig 5 should be for Fig 4, the caption for Fig 6 should be for Fig 5, the caption for Fig 7 should be for Fig 6, the caption for Fig 8 should be for Fig 7, and the caption for Fig 9 should be for Fig 8. The figure images appear in the correct order. The authors have provided a corrected version of the captions here.

**Fig 1:**
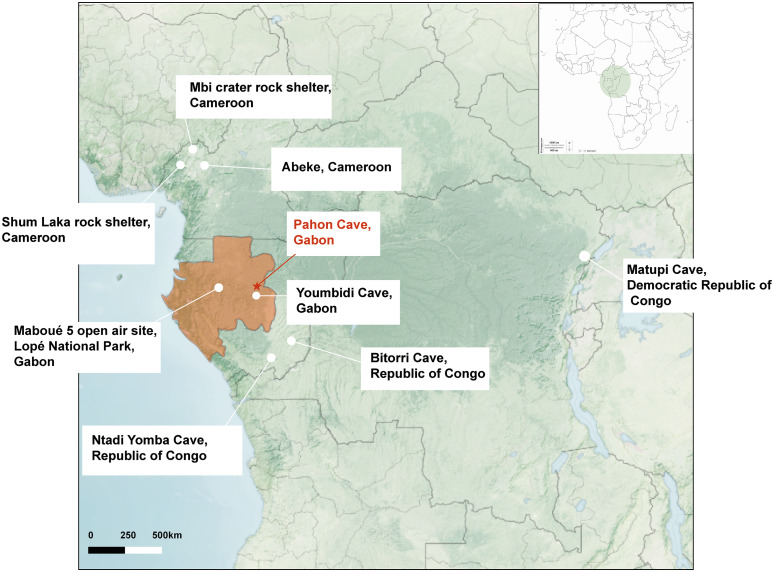
Map of Central Africa and the sites mentioned in the text. This Map shows the distribution of sites contemporany to Pahon sites. Map modified from https://earthobservatory.nasa.gov/map#5/-0.644/25.567‌‌.

**Fig 4:**
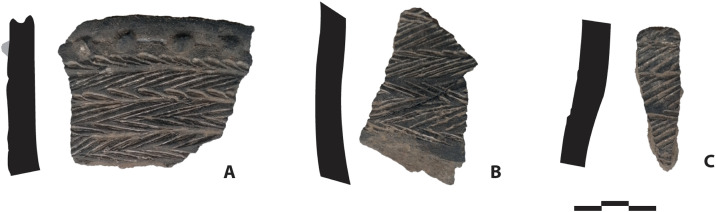
Pottery shards. Pottery sherds found on the surface of the Pahon Cave.

**Fig 5:**
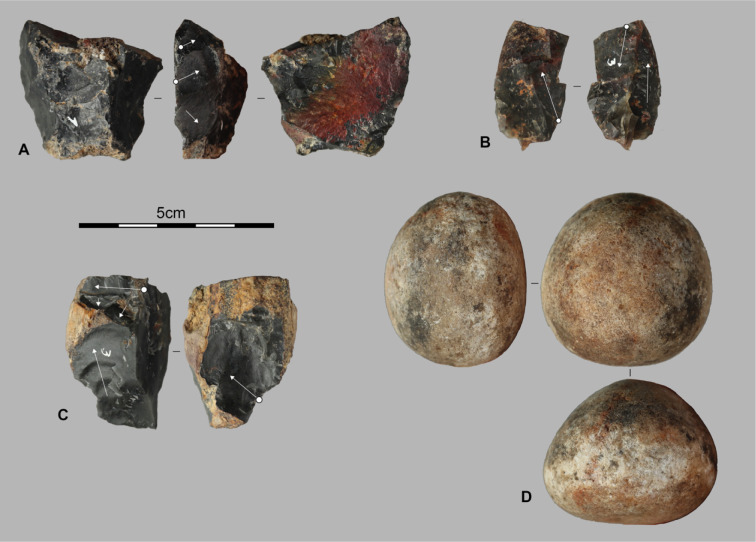
Lithic assemblages from the Pahon Cave. Artifacts from the 80–100 cm layer of the Pahon Cave; A and B are cores on flake, C is a core on a small block, and D is a hammerstone.

**Fig 6:**
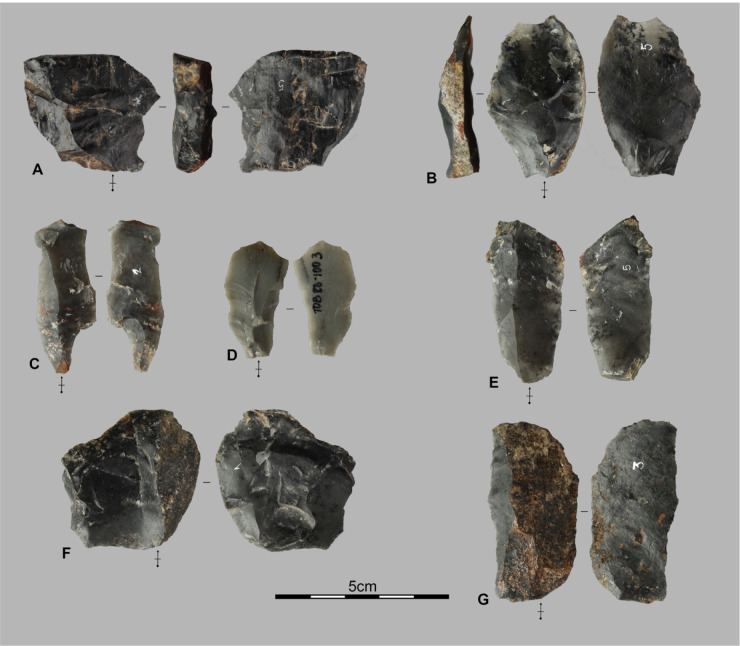
Lithic assemblages from the Pahon Cave. Artifacts from the 80–100 cm layer of the Pahon Cave; A and B are débordant flakes, C, D, E, and G are elongated flakes; F and G are semi-cortical flakes.

**Fig 7:**
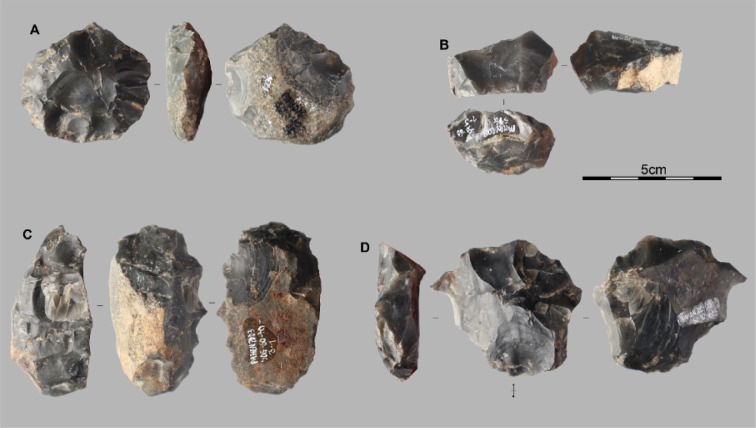
Lithic assemblages from the Pahon Cave. Examples of cores from Stratigraphic Unit 3: A is a centripetal core on a pebble, B on a block, C and D on flakes.

**Fig 8:**
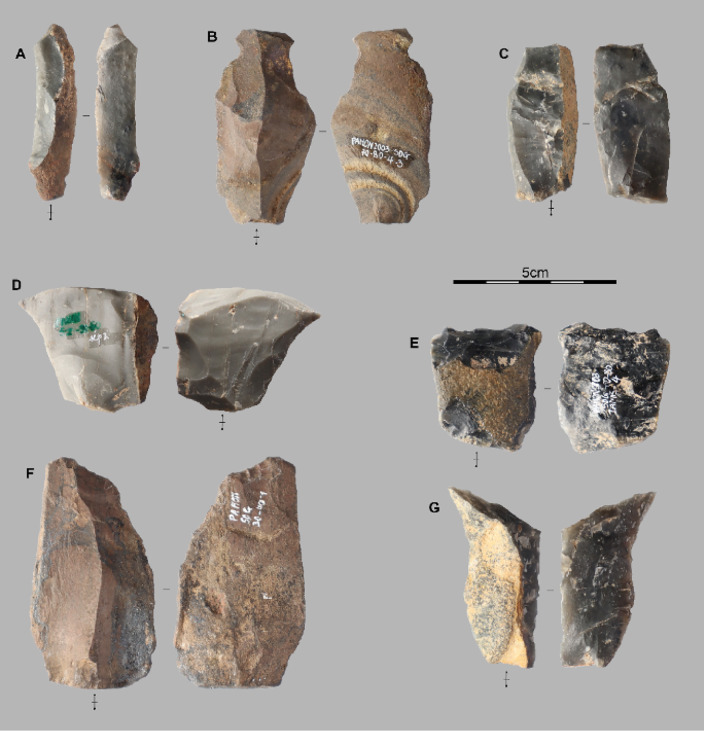
Lithic assemblages from the Pahon Cave. Flakes from Stratigraphic Unit3: A, B, and C are elongated flakes; D and F are retouched flakes; E and G are cortical and semi-cortical flakes.

**Fig 9:**
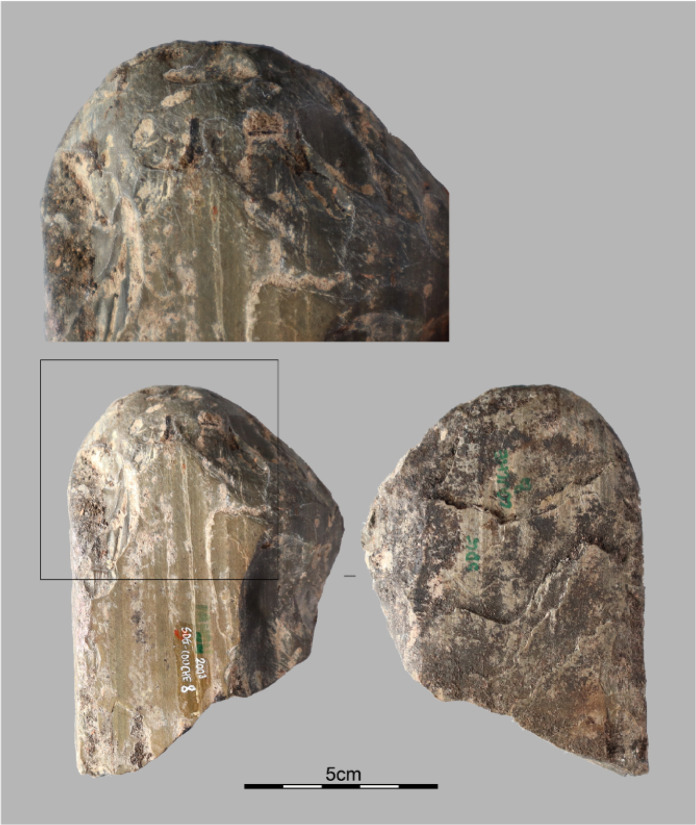
Fragment of a polished axe. Polished adze fragment from Stratigraphic Unit 3 dated between 6,604 and 5,660 cal BP.
